# The Proportion of Regulatory T Cells in Patients with Systemic Lupus Erythematosus: A Meta-Analysis

**DOI:** 10.1155/2018/7103219

**Published:** 2018-09-03

**Authors:** Sheng-Xiao Zhang, Xiao-Wen Ma, Yu-Feng Li, Na-Ling Lai, Ze-Hao Huang, Kai Fan, Cai-Hong Wang, Xiao-Feng Li

**Affiliations:** ^1^Department of Rheumatology, The Second Hospital of Shanxi Medical University, 382 Wuyi Road, Taiyuan, Shanxi 030001, China; ^2^Department of Hematology, The Second Hospital of Shanxi Medical University, 382 Wuyi Road, Taiyuan, Shanxi 030001, China; ^3^Department of Neurology, Shanxi Dayi Hospital Affiliated to Shanxi Medical University, 99 Longcheng Street, Taiyuan, Shanxi 030024, China

## Abstract

**Background:**

Accumulating evidence indicates that a deficiency in or dysfunction of regulatory T cells (Tregs) is involved in the pathogenesis of systemic lupus erythematosus (SLE). As different markers have been used to identify Tregs, recent studies on the proportions of Tregs in SLE patients have generated controversial results. To clarify the status of Tregs in such patients, we determined the proportions of Tregs present during development of the disease, with special consideration of controversial cellular markers.

**Methods:**

We identified studies reporting the proportions of Tregs in SLE patients by searching relevant databases through March 2018. Using the PRISMA guidelines, we performed a random effects meta-analysis of the frequencies of Tregs defined in different ways. Inconsistency was evaluated using the *I*-squared index (*I* ^2^), and publication bias was assessed by examining funnel plot asymmetry using the Begger and Egger tests.

**Results:**

Forty-four studies involving 2779 participants were included in the meta-analysis. No significant difference in the proportions of Tregs was evident between 1772 patients and 1007 controls [−0.191, (−0.552, 0.362), *p* = 0.613, *I* ^2^ = 95.7%]. We next conducted subanalyses based on individual definitions of Tregs. When the Treg definition included “FOXP3-positive” cells, the proportions did not differ between SLE patients and controls [−0.042, (−0.548, 0.632), *p* = 0.889, *I* ^2^ = 96.6%]; this was the case when Tregs were defined as either “CD25^low/−^FOXP3^+^” or “CD25^high/+^FOXP3^+^” cells. SLE patients had lower proportions of Tregs that were “single CD25-positive” [−1.428, (−1.982, −0.873), *p* < 0.001, *I* ^2^ = 93.4%] and “CD127-negative” [−1.093, (−2.002, −0.183), *p* = 0.018, *I* ^2^ = 92.6%] compared to controls. Tregs defined as “CD25^bright^,” “CD25^bright/high^CD127^low/−^,” and “CD25^high^CD127^low/−^FOXP3^+^” did not differ in proportion between SLE patients and controls.

**Conclusions:**

The Treg proportions varied by the cellular identification method used. The proportions of Tregs that were accurately identified and functionally validated fell among patients with SLE. Stricter definitions of Tregs are necessary when evaluating the status of such patients.

## 1. Introduction

Systemic lupus erythematosus (SLE) is a multisystem autoimmune disease characterized by highly variable clinical manifestations associated with widespread inflammation and overproduction of autoantibodies [1]. Growing evidence suggests that regulatory T cells (Tregs) maintain peripheral tolerance by controlling and limiting harmful immune responses [2]. Failure to maintain appropriate numbers of functional Tregs plays an important role in SLE pathogenesis [3].

However, initial studies of Treg status in the peripheral blood (PB) of patients with SLE have generated controversial results. One reason for the inconsistencies is that multiple phenotypes of Tregs are identified using different markers [3]. Tregs were first described as a peripheral CD4^+^ subpopulation expressing interleukin- (IL-) 2 receptor alpha chains (CD25) [4]. Further studies revealed that CD25 was expressed not only on Tregs but also on activated cells lacking regulatory functions, although the CD4^+^ T cell subset expressed the highest levels of CD25 (CD4^+^CD25^high^) and exhibited in vitro immunosuppressive features [5]. Forkhead box protein P3 (FOXP3), a transcription factor expressed at high levels in authentic Tregs, plays a key role in Treg development and is thought to be one of the most specific Treg cell markers [6]. However, the marker cannot be used to sort live cells as the protein is intracellular. In addition, CD127, the alpha chain of the IL-7 receptor, was reported to be upregulated on human T cells after activation and downregulated on Tregs [7], being inversely correlated with the FOXP3 expression level. Thus, costaining for CD127 and CD25 has been proposed to efficiently discriminate between Tregs and activated T cells [8]. The available data on the proportions and phenotypes of Tregs of SLE patients are contradictory; more studies are required to better understand the role played by Tregs during the disease course.

Here, we meta-analyze reports documenting the proportion of Treg cells among CD4^+^ T cells in the PB of patients with active and inactive SLE, as well as healthy controls, to better understand Treg malfunctions in patients with SLE.

## 2. Methods

### 2.1. Data Sources and Searches

This meta-analysis was performed as suggested by the Preferred Reporting Items for Systematic Reviews and Meta-Analyses (PRISMA) Statement and was registered at the International Prospective Register of Systematic Reviews (no. CRD42017060258) [9]. We searched for relevant studies published between January 1, 1950, and March 1, 2018, using PubMed, Embase, the Cochrane database, the Web of Knowledge, Clinical Trials.gov, and FDA.gov, with no restrictions in terms of the primary outcome or publication language. We used the MeSH terms “lupus erythematosus, systemic” and “T-lymphocytes, regulatory” and their combination. All potentially eligible studies were considered except for reviews and murine experiments. Key articles listed in the references were retrieved manually.

### 2.2. Study Selection and Data Extraction

The inclusion criteria were (1) evaluation of the proportion of Tregs among CD4^+^ T cells of SLE patients using the 1997 revised American College of Rheumatology criteria, (2) available as a full text article, and (3) information on the number of patients and controls. Two investigators independently selected and identified relevant publications, and a third investigator resolved any disagreements. The evidence levels of the studies were assessed based on the 2011 guidelines of the Oxford Center for Evidence-Based Medicine [10]. Quality assessment was done with the Newcastle-Ottawa Quality Assessment Scale, which can be used to assess the quality of nonrandomized studies [11].

We recorded patient baseline characteristics and their country of origin, the year of publication, the number of patients and controls, the definition of Tregs used (including CD25^+^, CD25^bright^, CD25^high^, CD25^low/−^FOXP3^+^, FOXP3^+^, CD25^+^FOXP3^+^, CD25^high^FOXP3^+^, CD25^+^CD127^−^, CD25^bright/high^CD127^low/−^, and CD25^high^CD127^low/−^FOXP3^+^), and the mean (or median) and standard deviation (SD) of the proportion of Tregs among CD4^+^ T cells. Data on the proportion of Tregs in patients with active and inactive SLE were also extracted.

### 2.3. Statistical Analysis

For continuous outcomes (the proportions of Tregs among CD4^+^ T cells of patients with active and inactive SLE and healthy controls), we calculated standardized mean differences (SMDs) and compared these values using a random effects model (REM) (the DerSimonian and Laird method) [12]. When Treg percentages were reported as medians with interquartile ranges (IQRs), we calculated means and SD (SD = IQR/1.35) [13]. The Cochrane chi-squared test was used to explore between-study heterogeneity. As heterogeneity was high (*I* ^2^ > 75%), we drew forest plots and performed subgroup analyses to explore the possible effects of study characteristics on outcomes. Publication bias was assessed by examining funnel plot asymmetry using the Begger and Egger tests (*p* ≥ 0.05). Preplanned sensitivity analysis was performed by omitting each study individually and calculating the remaining pooled effect. All statistical analyses were conducted using Stata software (ver. 12.0).

## 3. Results

### 3.1. Study Characteristics

**Figure 1:**
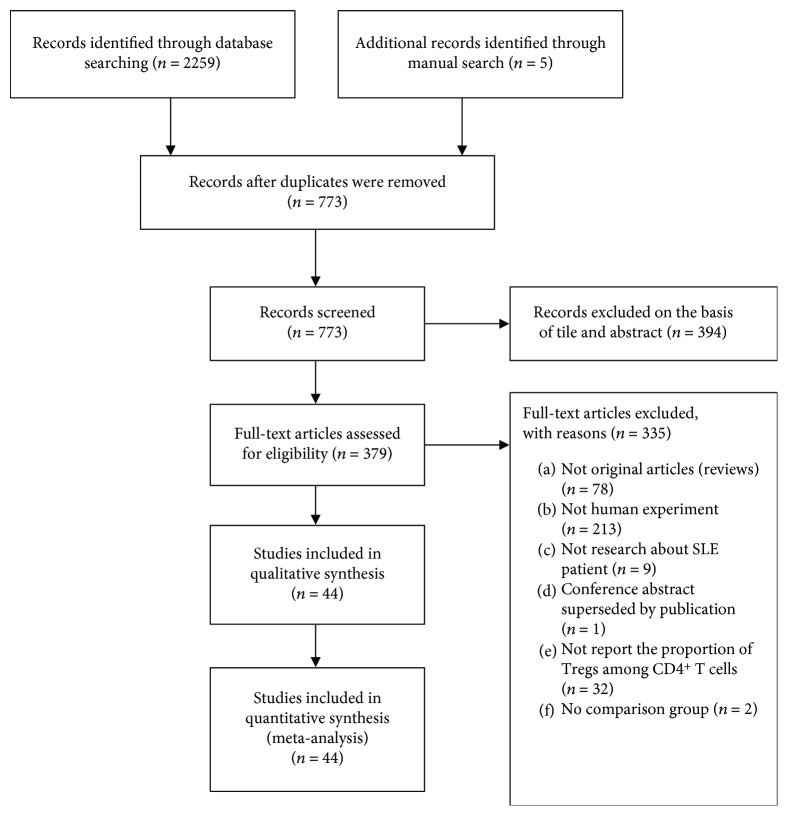
The study selection process.

We identified 2264 studies, of which 44 (with data on 1772 patients and 1007 controls) were included in analysis (Figure 1). The details are shown in Table 1. The average age of the SLE patients ranged from 8.7–45.4 years; the proportion of females ranged from 56.7–100%, the disease duration from 1.5–28.6 years, the average erythrocyte sedimentation rate from 18.6–78.8 mm/hour, and the SLE Disease Activity Index (SLEDAI) from 2.0–17.4. Patients were treated with corticosteroids (CS) and immunosuppressants including cyclophosphamide (CTX), azathioprine (AZA), cyclosporin A (CsA), mycophenolate mofetil (MMF), and chloroquine (HCQ). All controls were healthy without any autoimmune disease. All studies were poor-quality case-control studies or case series; thus, they were all of evidence level 4. We regarded all studies as case-control studies and scored them using the Newcastle-Ottawa Quality Assessment Scale (NOQAS); all studies had a score of 3–5.

### 3.2. Proportion of Tregs in the PB of SLE Patients

**Figure 2:**
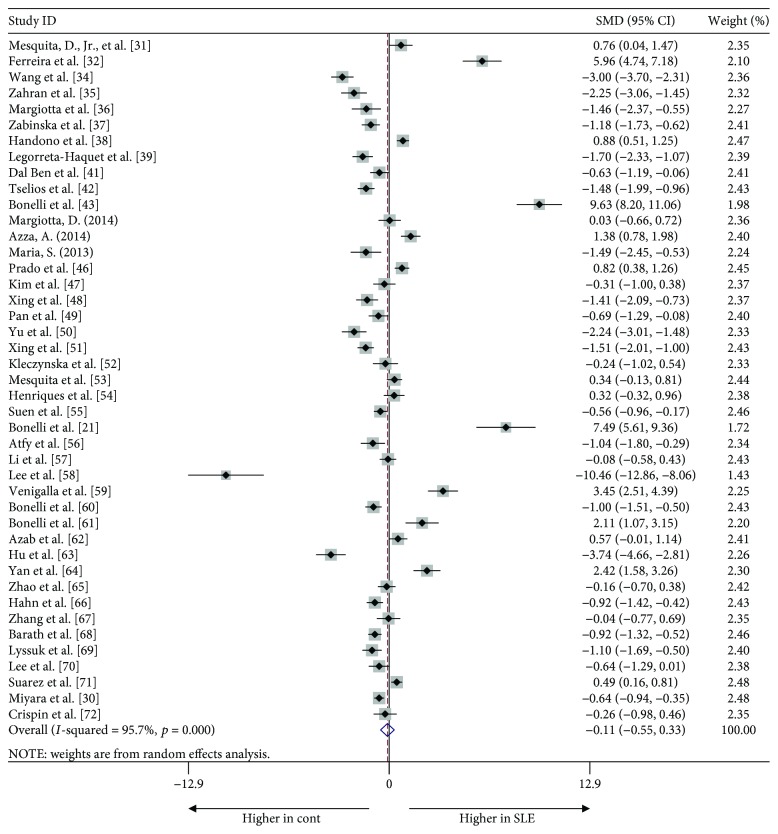
Forest plot of the overall meta-analysis of regulatory T cell (Treg) proportions in peripheral blood (PB), regardless of the Treg definitions used, between systemic lupus erythematosus (SLE) patients and healthy controls.

We initially compared the proportion of Tregs in SLE patients and healthy controls regardless of the Treg definition used. Surprisingly, no significant difference was apparent in any study [−0.113, (−0.552, 0.362), *p* = 0.613]. Also, heterogeneity, as assessed by the *I* ^2^ statistic, was 95.7% (*p* < 0.001) and thus very high. The Egger test revealed no publication bias (*t* = 0.70, *p* = 0.491) (Figure 2).

We hypothesized that the primary reason for the unexpected results might be that the definitions of Tregs were inconsistent. Thus, we performed subgroup analysis based on the Treg definitions to explore the potential sources of heterogeneity. First, we analyzed studies that identified Tregs only as “CD25-positive” (Supplementary Figure 1). Pooled analysis of all 18 trials revealed a significant decrease in the proportion of Tregs in SLE patients compared to controls [−1.428, (−1.982, −0.873), *p* < 0.001] with statistically significant between-study heterogeneity (*I* ^2^ = 93.4%, *p* < 0.001) and publication bias detected by the Egger test (*t* = −4.29, *p* = 0.001). In detail, we found significant differences in the proportion of Tregs between SLE patients and healthy controls when Tregs were defined as “CD25^+^” cells [−1.512, (−2.488, −0.535), *p* = 0.002] and as “CD25^high^” cells [−1.074, (−1.830, −0.318), *p* = 0.005]. However, in two studies, the proportion of Tregs defined as “CD25^bright^” cells did not differ significantly between patients and healthy controls [−3.495, (−9.197, 2.207), *p* = 0.230] (Table 2).

Second, we analyzed studies in which Tregs were defined as “FOXP3^+^” cells (Supplementary Figure 2). Pooled analysis of all 36 trials revealed no significant difference in the proportion of such Tregs between SLE patients and controls [0.042, (−0.548, 0.632), *p* = 0.889]. Statistically significant heterogeneity was evident among the studies (*I* ^2^ = 96.6%, *p* < 0.001). The Egger test detected no publication bias (*t* = 0.81, *p* = 0.424). Among the studies, five used “CD25^low/−^FOXP3^+^” to define Tregs, and three simply “FOXP3^+^”; the proportion of Tregs in SLE patients appeared to be higher than in controls [5.409, (2.112, 8.705), *p* = 0.001; 1.101, (0.435, 1.768), *p* = 0.001, resp.]. However, pooling of these data with those of other studies identifying Tregs as “CD25^high/+^FOXP3^+^” cells revealed a lower proportion of Tregs in patients than controls; Tregs were identified as “CD25^+^FOXP3^+^” cells [−1.279, (−2.079, −0.479), *p* = 0.00] and “CD25^high^FOXP3^+^” cells [−0.663, (−1.289, −0.036), *p* = 0.038] (Table 2).

Finally, the other eight groups that used “CD127-negative” to define Tregs showed that such cell numbers decreased in SLE patients [−1.093, (−2.002, −0.183), *p* = 0.018] with statistical heterogeneity (*I* ^2^ = 92.6%, *p* < 0.001) and publication bias (*t* = −3.05, *p* = 0.022). More specifically, pooling the data of four studies in which Tregs was identified as “CD25^+^CD127^−^” cells revealed a significant difference between SLE patients and controls [−1.093, (−2.002, −0.183), *p* = 0.018], but no significant difference was apparent when Tregs were defined as “CD25^bright/high^CD127^low/−^” cells [−12.392, (−37.922, 12.138), *p* = 0.341] or “CD25^high^CD127^low/−^FOXP3^+^” cells [−0.667, (−2.664, 1.331), *p* = 0.513] (Supplementary Figure 3 and Table 2).

As heterogeneity was apparent, we used a random effects model to prepare forest plots. We hypothesize that the significant heterogeneity might have been caused by differences in the experimental methods and clinical type and severity of disease among the different studies.

### 3.3. Disease Activity and the Proportion of Tregs in PB

**Figure 3:**
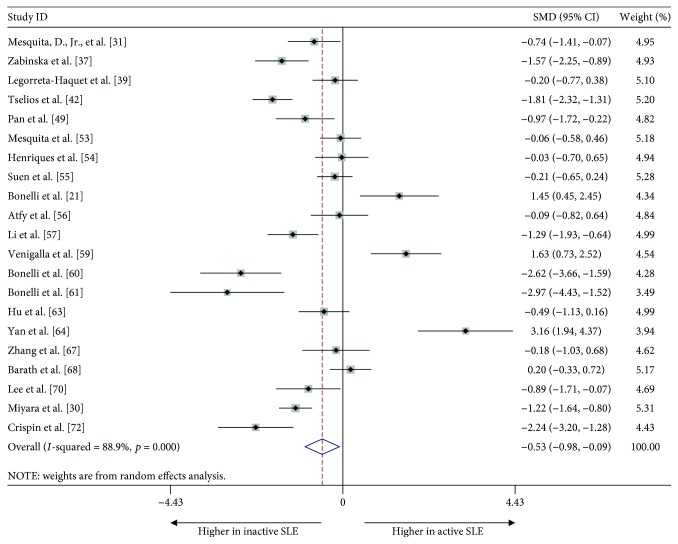
Forest plot of the overall meta-analysis of the proportion of Tregs in PB, regardless of the Treg definition used, in patients with active and inactive SLE.

To further assess the effect of disease activity, we analyzed 22 studies that reported the proportion of Tregs in active and inactive SLE patients, regardless of the Tregs definitions used. We found a significant reduction in the proportion of Tregs in patients with active compared to inactive disease [−0.520, (−0.976, −0.086), *p* = 0.019]. The heterogeneity, as assessed by the *I* ^2^ statistic, was 88.9% (*p* < 0.001) (Figure 3). No publication bias was evident in the Egger test (*t* = 0.52, *p* = 0.608).

## 4. Discussion

It is now widely accepted that the immune system includes Tregs that specialize in the maintenance of immune tolerance and homeostasis and secrete various immunosuppressive and anti-inflammatory cytokines, such as transforming growth factor-*β* (TGF-*β*), IL-10, IL-27, and IL-35 [14]. Treg deficiencies have been suggested to contribute to the immunological aberrations seen in SLE and other autoimmune diseases [3]. As Tregs exhibit multiple phenotypic features and express various markers, especially inconsistent markers were used to identify Treg cells in flow cytometry in previous studies, the proportion of Tregs in the PB of SLE patients has been controversial. To elucidate Treg status in such patients, we meta-analyzed their proportions relative to CD4^+^ T cells in SLE patients. As expected, the proportion of Tregs in patients with active SLE patients was significantly less than that in those with inactive SLE, suggesting that Treg cell depletion accelerated disease progression. However, the overall meta-analysis found no significant difference in Treg proportions between patients and healthy controls, although significant between-study heterogeneity was evident. We considered that the primary reasons for such unexpected results were due to inconsistent definitions of Tregs based on diverse markers used; thus, we subanalyzed the Treg data by the markers used for Treg identification, including CD25, FOXP3, and CD127.

Expression of CD25, the alpha chain of the IL-2 receptor [15], correlates positively with Treg functionality. The Treg-suppressive capacity is restricted to the CD4^+^ T cells that express the highest levels of CD25 [16]. We found out that SLE patients had a lower proportion of Tregs termed “single CD25-positive” compared to healthy controls. However, no such significance was evident when Tregs were defined as “CD25^bright^,” indicating that use of the surface marker CD25 alone is inadequate. In 2008, Han et al. [17] found out that CD25^high^ cells included a large proportion of FOXP3^−^ cells that could not be classified as Tregs. Other activated CD4^+^ T cells also express CD25 [16, 18], suggesting that more precise markers are needed to identify Tregs.

FOXP3 is a crucial regulator of Treg gene expression, being required for both Treg generation and survival [19]. Scurfy (Sf) mice with Treg abnormalities harbor a missense mutation in FOXP3 [6] and develop anti-dsDNA, anti-Smith, and antinuclear antibodies similar to those of SLE patients. Such FOXP3 mutant mice also exhibit multiorgan inflammation of systems usually involved in SLE [20]. However, when Tregs were defined as “FOXP3-positive” cells, the proportions of such cells did not differ between SLE patients and controls because the definitions of Tregs were complicated by the addition of CD25 status, giving “CD25-negative and FOXP3-positive” and “CD25 and FOXP3 double positive.” This phenomenon may be explained by the findings of other studies indicating that the CD4^+^CD25^−^FOXP3^+^ T cells of SLE were dysfunctional Tregs [21, 22] and may even be previously activated conventional T cells [23].

To distinguish Tregs from conventional CD4^+^ T cells, the inclusion of additional markers, such as CD127, has been proposed, because Tregs express low levels of this protein (whereas activated T cells express high levels). We found a lower proportion of CD127-negative Tregs in SLE patients compared with healthy controls, suggesting that CD127 combined with other markers could be used to identify Tregs.

Further, the controversial status of Tregs in PB of SLE patients might also be related to the different subsets of Tregs. Tregs can be classified into nTregs [24], iTregs [25], Tr1 cells [26], Th1-like, Th2-like, or Th17-like Tregs [27], and so forth. One of the largest Treg subsets is nTregs, which are developed from the thymus and express CD4, CD25, and FOXP3 [24]. In contrast to nTregs, iTregs are generated in the periphery and induced to express FOXP3 in response to foreign antigens that are much intrinsically unstable in inflammatory compared to nTregs [25]. Interestingly, inflammatory conditions of stimulation can skew nTreg differentiation to Tr1 cells in active lupus [28]. Tr1 cells are another subset of CD4^+^ T cells in the absence of FOXP3 expression characterized by the ability to secrete IL-10 and inhibit T cell responses by disrupting the metabolic state of T effector cells [26]. Although there are important differences of these cells, there is no definitive protein markers that effectively distinguishes among all these Treg cell populations in vitro or in vivo. To date, it is still challenging to value the real status of above Treg subsets in patients with SLE.

The limitations of our work include the fact that we did not consider disease duration or treatment, as both the drugs used and disease staging were inconsistent; however, these factors might affect the proportion of Tregs in PB. Additionally, disease activity was scored differently among studies; some regarded active SLE to be present when the SLEDAI was ≥6, but others used different cutoffs; these differences may have influenced the results. Moreover, Tregs are usually evaluated in PB, in which tissue Treg cell status may fluctuate [3]. Also, information on Treg aberrations in lymphoid tissues or at sites of active disease, for example, the skin of patients with cutaneous lupus [29] or the kidneys of patients with active glomerulonephritis [30], was lacking.

## 5. Conclusion

In conclusion, we suggest that the reported variations of Treg status among SLE patients are attributable to inconsistent Treg identification; different markers are employed. Here, we analyzed the effects of the use of such markers on the reported proportion of Tregs. Our findings lend support to the idea that the Treg status of SLE patients is important, but we could not determine the best definition of Tregs. Further studies are needed on the definition and function of Tregs.

## Figures and Tables

**Table 1:** Characteristics of the individual studies included in the meta-analysis.

Author (ref.)	Publish year	Country	EL^a^	Q^b^	Case numbers		Tregs' definition	Data type	% of Tregs among CD4^+^ T cells [mean (or median) ± SD]	
					SLE	HC			SLE	HC
Mesquita, D., Jr., et al. [31]	2018	Brazil	4	4	37	10	CD4^+^FOXP3^+^	Calculated	6.49 ± 4.99	3.05 ± 1.76
Ferreira et al. [32]	2017	UK	4	5	34	24	CD25^low^CD127^low^FOXP3^+^	Calculated	18.8 ± 2.64	6.39 ± 0.74
Singla et al. [33]	2017	USA	4	4	2	1	CD25^+^FOXP3^+^	Calculated	4.48 ± 0.49	2.97
							CD25^high^FOXP3^high^	—	1.02 ± 0.97	0.58
Wang et al. [34]	2017	China	4	5	47	25	CD25^+^FOXP3^+^	Calculated	2.32 ± 0.15	3.02 ± 0.34
Zahran et al. [35]	2016	Egypt	4	4	20	20	CD25^+/high^FOXP3^+^	Original	1.32 ± 0.25	2.24 ± 0.52
Margiotta et al. [36]	2016	Italy	4	3	13	11	CD25^high^FOXP3^+^	Original	1.27 ± 0.9	2.8 ± 1.2
Zabinska et al. [37]	2016	Poland	4	4	54	19	CD25^+^FOXP3^+^	Calculated	1.10 ± 1.27	3.36 ± 0.52
							CD25^+^CD127^−^	Calculated	5.79 ± 2.13	8.11 ± 1.4
Handono et al. [38]	2016	Indonesia	4	5	62	62	CD25^+^FOXP3^+^	Original	2.3 ± 2.1	0.9 ± 0.8
Legorreta-Haquet et al. [39]	2016	Mexico	4	3	47	17	CD25^high^CD127^low/−^FOXP3^+^	Calculated	1.54 ± 0.84	2.92 ± 0.73
Eltayeb et al. [40]	2014	Egypt	4	3	37	20	CD25^bright^FOXP3^+^	Original	52.6 ± 4.2	45.6 ± 6.4
Dal Ben et al. [41]	2014	Brazil	4	4	25	25	CD25^+^FOXP3^+^	Original	0.74 ± 0.34	1.83 ± 0.77
							CD25^+^	Original	1.28 ± 0.89	1.81 ± 0.8
Tselios et al. [42]	2014	Greece	4	4	100	20	CD25^high^FOXP3^+^	Calculated	0.99 ± 0.36	1.49 ± 0.19
Bonelli et al. [43]	2014	Austria	4	4	61	36	CD25^−^FOXP3^+^	Original	5.1 ± 0.5	1.1 ± 0.2
Szmyrka-Kaczmarek et al. [44]	2014	Poland	4	4	21	13	CD25^high^FOXP3^+^	Original	18.57 ± 10.44	32.08 ± 11.54
							CD25^+^	Original	6.75 ± 3.73	6.65 ± 1.59
Longhi et al. [45]	2013	United Kingdom	4	3	12	10	CD25^+^CD127^−^	Original	88.9 ± 3.2	92.5 ± 0.7
							CD25^+^FOXP3^+^	Original	9.27 ± 2.2	19.9 ± 2.5
							CD25^high^CD127^−^	Original	89.5 ± 0.5	99.2 ± 0.1
							CD25^high^FOXP3^+^	Original	1.07 ± 0.37	2.04 ± 0.17
Prado et al. [46]	2013	Spain	4	3	75	29	CD25^high^FOXP3^+^	Calculated	1.65 ± 1.41	1.47 ± 0.68
							FOXP3^+^	Calculated	10.22 ± 5.10	6.41 ± 3.13
							CD25^−^FOXP3^+^	Calculated	6.71 ± 5.37	3.13 ± 1.61
Kim et al. [47]	2012	Korea	4	3	13	22	CD25^high^FOXP3^+^	Original	4.6 ± 1.3	5.0 ± 1.3
Xing et al. [48]	2012	China	4	4	20	22	CD25^+^FOXP3^+^	Original	5.12 ± 0.67	6.12 ± 0.74
							CD25^high^	Original	2.08 ± 0.32	2.76 ± 0.49
Pan et al. [49]	2012	China	4	3	41	15	CD25^+^FOXP3^+^	Calculated	3.44 ± 0.74	3.90 ± 0.40
Yu et al. [50]	2012	China	4	4	16	30	CD25^+^FOXP3^+^	Original	1.53 ± 0.8	3.97 ± 1.21
							CD25^+^CD127^−^		2.46 ± 1.12	4.43 ± 1.05
Xing et al. [51]	2012	China	4	4	60	28	CD25^+^FOXP3^+^	Calculated	4.57 ± 1.07	6.09 ± 0.86
Kleczynska et al. [52]	2011	Poland	4	4	15	11	CD25^high^FOXP3^+^	Calculated	1.80 ± 1.56	2.10 ± 0.67
Mesquita et al. [53]	2011	Brazil	4	4	57	26	CD25^high^CD127^low/−^FOXP3^+^	Calculated	0.97 ± 1.04	0.66 ± 0.50
Henriques et al. [54]	2010	Portugal	4	4	34	13	CD25^bright^CD127^low/−^	Calculated	8.16 ± 3.53	7.10 ± 2.70
Suen et al. [55]	2009	China	4	4	87	36	CD25^high^FOXP3^+^	Calculated	0.64 ± 0.39	0.86 ± 0.39
Bonelli et al. [21]	2009	Austria	4	4	22	15	CD25^−^FOXP3^+^	Original	7.5 ± 1.0	1.4 ± 0.4
Atfy et al. [56]	2009	Egypt	4	4	30	10	CD25^high^	Calculated	6.18 ± 1.90	8.07 ± 2.04
							CD25^+^	Calculated	15.29 ± 5.97	21.3 ± 5.0
Li et al. [57]	2009	China/Chinese	4	4	47	22	CD25^+^FOXP3^+^	Calculated	3.37 ± 1.83	3.5 ± 1.4
Lee et al. [58]	2008	Korea	4	3	20	21	CD25^+^	Original	15.2 ± 0.2	22.1 ± 0.9
Venigalla et al. [59]	2008	Germany	4	4	26	19	CD25^low^FOXP3^+^	Calculated	10.68 ± 1.63	6.3 ± 0.4
							CD25^high^FOXP3^+^	Calculated	2.35 ± 0.51	1.75 ± 0.10
Bonelli et al. [60]	2008	Austria	4	3	58	24	CD25^high^	Calculated	1.15 ± 1.00	2.0 ± 0.1
Bonelli et al. [61]	2008	Austria	4	3	17	8	CD25^high^	Calculated	1.06 ± 0.40	1.8 ± 0.16
							FOXP3^+^	Calculated	13.02 ± 3.60	6.5 ± 1.3
Azab et al. [62]	2008	Egypt	4	4	24	24	CD25^+^	Original	10.37 ± 4.44	7.78 ± 4.69
Hu et al. [63]	2008	China	4	4	38	16	CD25^+^	Calculated	4.91 ± 2.97	16.25 ± 3.19
Yan et al. [64]	2008	China	4	3	25	15	CD25^+^FOXP3^+^	Calculated	8.00 ± 1.64	4.78 ± 0.43
Zhao et al. [65]	2008	China/Chinese	4	3	29	24	CD25^+^FOXP3^+^	Original	2.1 ± 1.2	4.0 ± 1.4
							CD25^+^CD127^−^	Original	4.7 ± 2.3	5.0 ± 1.2
							CD25^high^	Original	0.8 ± 0.4	1.8 ± 0.8
Hahn et al. [66]	2008	America	4	4	36	32	CD25^high^	Calculated	1.24 ± 0.50	1.85 ± 0.81
Zhang et al. [67]	2008	China	4	4	21	11	CD25^+^FOXP3^+^	Calculated	4.51 ± 3.30	4.68 ± 5.77
Barath et al. [68]	2007	Hungary	4	4	72	41	CD25^high^FOXP3^+^	Original	3.06 ± 1.45	4.26 ± 1.01
Lyssuk et al. [69]	2007	Russia	4	3	43	17	CD25^+^FOXP3^+^	Original	1.8 ± 0.8	4.9 ± 1.4
							CD25^+^	Original	6.1 ± 3.8	10.3 ± 3.9
Lee et al. [70]	2006	Taiwan	4	3	27	15	CD25^+^	Calculated	8.13 ± 2.80	9.78 ± 2.11
Suarez et al. [71]	2006	Spain	4	3	110	56	CD25^high^	Original	8.34 ± 7.04	5.47 ± 2.43
Miyara et al. [30]	2005	France	4	5	107	82	CD25^bright^	Calculated	0.95 ± 0.62	1.29 ± 0.38
Crispin et al. [72]	2003	Mexico	4	3	30	10	CD25^+^	Calculated	18.6 ± 8.18	20.6 ± 5.9
							CD25^bright^	Calculated	1.6 ± 0.04	2.57 ± 0.3

SLE: systemic lupus erythematosus. ^a^Evidence level (EL) of each study was based on Oxford Center for Evidence-Based Medicine 2011. ^b^Quality (Q) of each study was based on the Newcastle-Ottawa Quality Assessment Scale case.

**Table 2:** Subgroup analysis based on different definitions of Tregs in PB of patients with SLE.

Definition of Tregs	Number of studies	Test of association			Test of heterogeneity		Egger's test	
		SMD^a^	95% CI	*p* value	*I* ^2^	*p* value	*t*	*p*
*Single CD25-positive*	18	−1.428	(−1.982, −0.873)	<0.001	93.4	<0.001	−4.29	0.001
CD25^+^	9	−1.512	(−2.488, −0.535)	0.002	93.9	<0.001	−4.20	0.004
CD25^bright^	2	−3.495	(−9.197, 2.207)	0.230	97.9	<0.001	—	—
CD25^high^	7	−1.074	(−1.830, −0.318)	0.005	92.0	<0.001	−4.28	0.008
*Associated with FOXP3-positive*	29	−0.043	(−0.641, 0.554)	0.887	96.3	<0.001	0.55	0.585
CD25^low/−^FOXP3^+^	4	5.275	(1.415, 9.136)	0.007	98.3	<0.001	4.05	0.056
FOXP3^+^	2	1.377	(0.124, 2.631)	0.031	80.1	0.025	—	—
CD25^+^FOXP3^+^	13	−1.142	(−1.942, −0.341)	0.005	95.2	<0.001	−2.01	0.069
CD25^high^FOXP3^+^	10	−0.701	(−1.283, −0.118)	0.018	89.6	<0.001	−0.63	0.544
*Associated with CD127-negative*	8	−1.093	(−2.002, −0.183)	0.018	92.6	<0.001	−3.05	0.022
CD25^+^CD127^−^	4	−1.128	(−1.894, −0.361)	0.004	81.0	0.001	−1.12	0.379
CD25^bright/high^CD127^low/−^	2	−12.392	(−37.922, 12.138)	0.341	97.5	<0.001	—	—
CD25^high^CD127^low/−^FOXP3^+^	2	−0.667	(−2.664, 1.331)	0.513	96.1	<0.001	—	—

PB: peripheral blood; SLE: systemic lupus erythematosus; SMD: standard mean difference; CI: confidence interval; *I* ^2^: *I*-squared index. ^a^Magnitude of Cohen's *d* effect size (SMD): 0.2–0.5, small effect; 0.5–0.8, medium effect; ≥0.8, large effect.

## Supplementary Materials

Supplementary Materials Supplementary Table 1: background of SLE patients in each study. Supplementary Table 2: characteristics of the included studies measuring disease activity in patients with SLE. Supplementary Figure 1: forest plot of subgroup analyses of the proportion of Tregs, defined in terms of CD25-positivity (alone), among CD4^+^ T cells in PB. (a) Standardized mean differences (SMDs) (the proportion of Tregs [“CD25^+^” cells] among CD4^+^ T cells in the PB of SLE patients minus that of control subjects) as estimated by meta-analysis. (b) SMDs when Tregs were defined as “CD25^bright^” cells. (c) SMDs when Tregs were defined as “CD25^high^” cells. Supplementary Figure 2: forest plots of subgroup analyses of the proportion of Tregs defined as FOXP3-positive among CD4^+^ T cells in PB. (a) SMDs (the proportion of Tregs [“CD25^low/−^FOXP3^+^” cells] among CD4^+^ T cells in the PB of SLE patients minus that of control subjects) as estimated by meta-analysis. (b) SMDs when Tregs were defined as “FOXP3^+^” cells. (c) SMDs when Tregs were defined as “CD25^+^FOXP3^+^” cells. (d) SMDs when Tregs were defined as “CD25^high^FOXP3^+^” cells. Supplementary Figure 3: forest plots of the subgroup analyses of the proportion of Tregs that were CD127-negative among PB CD4^+^ T cells. (a) SMDs (the proportion of Tregs [“CD25^+^CD127^−^” cells] among CD4^+^ T cells in the PB of SLE patients minus that of control subjects) as estimated by meta-analysis. (b) SMDs when Tregs were defined as “CD25^high^CD127^low/−^” cells. (c) SMDs when Tregs were defined as “CD25^high^CD127^−^” cells. (d) SMDs when Tregs were defined as “CD25^high^CD127^low/−^FOXP3^+^” cells. Supplementary Figure 4: funnel plot for publication bias in an association analysis of Treg proportions in PB, regardless of the Treg definitions used, between systemic lupus erythematosus (SLE) patients and healthy controls. Supplementary Figure 5: funnel plot for publication bias in an association analysis of the proportion of Tregs, defined in terms of CD25-positivity (alone), among CD4^+^ T cells in PB. Supplementary Figure 6: funnel plot for publication bias in an association analysis of the proportion of Tregs defined as FOXP3-positive among CD4^+^ T cells in PB. Supplementary Figure 7: funnel plot for publication bias in an association analysis of the proportion of Tregs that were CD127-negative among PB CD4^+^ T cells.
