# Supplementary material for: The Proportion of Regulatory T Cells in Patients with Systemic Lupus Erythematosus: A Meta-Analysis
Source: J Immunol Res. 2018 Sep 3;2018:7103219. doi: 10.1155/2018/7103219 (PMC6140280; doi:10.1155/2018/7103219)

| **Supplementary Table 1:** Background of SLE patients in each study | | | | | | | | |
| --- | --- | --- | --- | --- | --- | --- | --- | --- |
| Author (Ref.) | Publish Year | Age (years) | Female (%) | Disease duration (years) | SLEDAI | CRP (mg/d) | ESR (mm/hour) | Treatment |
| Ferreira, RC., et al.(27) | 2017 | 35±8.1 | 77.70% | 6.2±5.6 | NA | NA | NA | CTX,MMF |
| Singla, S., et al.(28) | 2017 | 41.15±15.98 | 94.10% | NA | NA | NA | NA | NA |
| Wang, X., et al.(29) | 2017 | 9±9 | 100% | NA | 7±7 | NA | NA | ASA, HCQ, IVP, MTX, CS, MMF |
| Zahran, A.M., A., et al.(30) | 2016 | 30.17±11.11 | 100% | NA | NA | NA | NA | NA |
| Margiotta, D., et al.(31) | 2016 | 9.4±3.7 | 90% | NA | 8.3±4.1 | 3.68±0.2 | 77.68±10.12 | royal jelly |
| Zabinska, M., et al.(32) | 2016 | 45.3±10.8 | 100% | 7.6±3.0 | 5.7±2.5 | 5.3±3 | 18.6±5.2 | NA |
| Handono, K., et al.(33) | 2016 | 36.5±13.7 | 96.30% | NA | 11.4±7.75 | NA | NA | CS, CTX, AZA, CSA A, MMF, HCQ |
| Legorreta-Haquet, M.V., et al.(34) | 2016 | 31.2±7.3 | NA | NA | >3 | NA | NA | vitamin A |
| Azza, A., A., et al.(35) | 2014 | 25±50 | NA | NA | NA | NA | NA | NA |
| Dal Ben, E.R., et al.(36) | 2014 | 8.7±4.5 | 56.70% | 2.68±3.38 | 12.56±5.5 | NA | NA | CS, AZA |
| Tselios, K., et al.(37) | 2014 | 43.5±12.84 | 96% | NA | 10±5.77 | NA | NA | NA |
| Bonelli, M. et al.(38) | 2014 | 45.4±13.9 | 89% | 9.03±8.46 | NA | NA | NA | HCQ, CS, MMF, AZA, CTX, |
| Szmyrka-Kaczmarek, M ., et al.(39) | 2014 | 40.4±14.5 | 91.80% | 5.4±4.9 | 4.2±4.2 | NA | NA | CS, HCQ, MMF, AZA, CTX, MTX |
| Longhi, M.S., et al.(40) | 2013 | 44.54±13.71 | 95% | 8.44±6.35 | 6.92±5.61 | NA | NA | NA |
| Prado, C., et al.(41) | 2013 | 34.57±13.43 | 91.67% | NA | NA | NA | NA | NA |
| Kim, J.R., et al.(42) | 2012 | 35.36±14.3 | 96% | 12.4±8.91 | ≥8 | NA | NA | NSAIDs, AM, CS, DMARDs |
| Xing, Q., et al.(43) | 2012 | 42±16.1 | 100% | NA | NA | NA | NA | NA |
| Pan, X., et al.(44) | 2012 | 28.4±4.6 | 95% | 1.9±1.6 | 12.6±8.2 | NA | NA | NA |
| Yu, N., et al.(45) | 2012 | 32.7±7.4 | NA | NA | 5.03±4.48 | NA | NA | not receiving drugs |
| Xing, Q., et al.(46) | 2012 | 30.25±10.64 | 100% | NA | NA | NA | NA | NA |
| Kleczynska, W., et al.(47) | 2011 | 34.4±7.6 | 96.70% | 8.6±7.9 | 12.8±10.2 | 12.4±7.4 | 27±11.2 | NA |
| Mesquita, D., et al.(48) | 2011 | 36.5±12.03 | 98% | 3.36±3.48 | 4.64±5.68 | NA | NA | CTX, CS, HCQ, |
| Henriques, A., et al.(49) | 2010 | 33.12±9.83 | 88.20% | 9±5 | 6.05±4.61 | NA | NA | HCQ, CS, MMF, AZA or CSA |
| Suen, J.L., et al.(50) | 2009 | 37.3±11.1 | 90.80% | NA | 5.5±5.2 | NA | NA | CS, HCQ, AZA |
| Bonelli, M., et al.(21) | 2009 | 45±16.8 | NA | NA | NA | NA | NA | NA |
| Atfy, M., et al.(51) | 2009 | 36.06±5.1 | 90% | 3.96±0.99 | 5.08±4.03 | NA | 78.8±14.9 | NA |
| Li, D.M., et al.(52) | 2009 | 33.23±12.19 | 85% | NA | NA | NA | NA | CS |
| Lee, H.Y., et al.(53) | 2008 | 31.8±11.34 | 85% | 6.9±4.29 | NA | NA | NA | NA |
| Venigalla, R.K., et al.(54) | 2008 | 40.57±14.54 | NA | NA | NA | NA | NA | NA |
| Bonelli, M., et al.(55) | 2008 | NA | NA | NA | NA | NA | NA | NA |
| Bonelli, M., et al.(56) | 2008 | NA | NA | NA | NA | NA | NA | NA |
| Azab, N.A., et al.(57) | 2008 | 24.46±6.48 | 83.30% | 3.2±2.48 | 17.42±10.51 | NA | NA | CS, HCQ, CTX |
| Hu, S., et al.(58) | 2008 | 31.5±11.2% | 92.10% | 3.3±1.4 | NA | NA | NA | NA |
| Yan, B., et al.(59) | 2008 | 25.7±9.6 | 100% | NA | (15)11.8±3.9;(10)<3 | NA | NA | CS |
| Zhao, S.S., et al.(60) | 2008 | 34±13 | 89.70% | 1.5 | 6--25 | NA | NA | NA |
| Hahn, B.H., et al.(61) | 2008 | 43.4±16.7 | 97% | 18.1±11 | 5±3.1 | NA | NA | CS, HCQ, MMF, AZA, CTX, MTX |
| Zhang, B., et al.(62) | 2008 | 32.8±12.3 | 90.50% | 28.6±11.8 | NA | NA | NA | CS, CTX |
| Barath, S., et al.(63) | 2007 | 34.4±13.9 | 87.50% | 9.4±8.1 | NA | NA | NA | CS, DMARDs |
| Lyssuk, E.Y., et al.(64) | 2007 | 30.47±10.48 | 81.40% | NA | NA | NA | NA | CS, CTX |
| Lee, J.H., et al.(65) | 2006 | 13.37±3.63 | 81.50% | NA | 4.62±2.73 | NA | NA | CS, HCQ, AZA |
| Suarez, A., et al.(66) | 2006 | 30.01±12.13 | 94.50% | NA | NA | NA | NA | CS, NSAIDs, DMARDs |
| Miyara, M., et al.(25) | 2005 | 35.8±14.5 | 91.60% | NA | NA | NA | NA | CS, HCQ, MTX, CTX |
| Crispin, J.C., et al.(67) | 2003 | NA | NA | NA | NA | NA | NA | NA |

Patient characteristics of SLE patients in each study. CRP = C-reactive protein; ESR = erythrocyte sedimentation rate; NA = not applicable; CS = CorticoCS; DMARDs = disease modified anti-rheumatic-drugs; CTX = Cyclophosphamide; MTX = methotrexate; HCQ = hydroxychloroquine; AZA = azathioprine; CSA = cyclosporin A;

| **Supplementary Table 2:** Characteristics of the included studies measuring disease activity in patients with SLE. | | | | | | | | |
| --- | --- | --- | --- | --- | --- | --- | --- | --- |
| Author (Ref.) | Publish year | Country | Case numbers | | Data type | % of Tregs among CD4^+^T cells [mean (or median) ± SD] | | Tregs’ definition |
|  |  |  | Active | Inactive |  | Active | Inactive |  |
| Mesquita, D., Jr., et al.(26) | 2018 | Brazil | 17 | 20 | Calculated | 4.6±3.22 | 8.09±5.70 | CD4^+^ FOXP3^+^ |
| Singla, S., et al.(28) | 2017 | USA | 1 | 1 | Original | 3.99 | 4.97 | CD4^+^CD25^+^ FOXP3^+^ |
|  |  |  |  |  |  | 0.058 | 1.99 | CD4^+^CD25^high^ FOXP33^high^ |
| Zabinska, M., et al.(32) | 2016 | Poland | 40 | 14 | Calculated | 1.02±0.6 | 2.31±1.3 | CD25^+^FOXP3^+^ |
| Legorreta-Haquet, M.V., et al.(34) | 2016 | Mexico | 25 | 22 | Calculated | 1.46±0.9 | 1.63±0.8 | CD25^high^CD127^low/-^FOXP3^+^ |
| Tselios, K., et al.(37) | 2014 | Greece | 28 | 72 | Calculated | 0.62±0.2 | 1.13±0.3 | CD25^high^FOXP3^+^ |
| Pan, X., et al.(44) | 2012 | China | 15 | 16 | Original | 3.00±0.9 | 3.70±0.5 | CD25^+^FOXP3^+^ |
| Mesquita, D., et al.(48) | 2011 | Brazil | 26 | 31 | Original | 0.94±0.8 | 1.00±1.2 | CD25^high^CD127^low/-^FOXP3^+^ |
| Henriques, A., et al.(49) | 2010 | Portugal | 15 | 19 | Original | 8.10±3.7 | 8.20±3.5 | CD25^bright^CD127l^ow/-^ |
| Suen, J.L., et al. (50) | 2009 | China | 58 | 29 | Original | 0.61±0.4 | 0.69±0.3 | CD25^high^FOXP3^+^ |
| Bonelli, M., et al. (21) | 2009 | Austria | 7 | 15 | Original | 10.50±5.4 | 6.10±0.8 | CD25^-^FOXP3^+^ |
| Atfy, M., et al. (51) | 2009 | Egypt | 12 | 18 | Original | 14.97±6.6 | 15.50±5.7 | CD25^high^ |
| Li, D.M., et al. (52) | 2009 | China/Chinese | 29 | 18 | Original | 2.60±1.1 | 4.60±2.1 | CD25^+^FOXP3^+^ |
| Venigalla, R.K., et al. (54) | 2008 | Germany | 14 | 12 | Original | 2.65±0.4 | 2.00±0.4 | CD25^high^FOXP3^+^ |
| Bonelli, M., et al. (55) | 2008 | Austria | 5 | 53 | Original | 0.96±0.1 | 1.17±0.1 | CD25^high^ |
| Bonelli, M., et al. (56) | 2008 | Austria | 6 | 11 | Original | 0.63±0.1 | 1.30±0.3 | CD25^high^ |
| Hu, S., et al. (58) | 2008 | China | 20 | 18 | Original | 4.24±3.1 | 5.66±2.7 | CD25^+^ |
| Yan, B., et al. (59) | 2008 | China | 15 | 10 | Original | 9.11±0.7 | 6.32±1.1 | CD25^+^FOXP3^+^ |
| Zhang, B., et al. (62) | 2008 | China | 11 | 10 | Calculated | 4.19±3.6 | 4.79±3.2 | CD25^+^FOXP3^+^ |
| Barath, S., et al. (63) | 2007 | Hungary | 19 | 53 | Original | 3.27±1.9 | 2.98±1.3 | CD25^high^FOXP3^+^ |
| Lee, J.H., et al. (65) | 2006 | Taiwan | 17 | 10 | Original | 7.27±2.5 | 9.59±2.8 | CD25^+^ |
| Miyara, M., et al.(25) | 2005 | France | 45 | 62 | Original | 0.57±0.2 | 122±0.7 | CD25^high^ |
| Crispin, J.C., et al. (67) | 2003 | Mexico | 10 | 20 | Original | 10.2±3.8 | 22.8±6.3 | CD25^+^ |

**Supplementary Fig. 1:** Forest plot of subgroup analyses of the proportion of Tregs, defined in terms of CD25-positivity (alone), among CD4+ T cells in peripheral blood (PB).


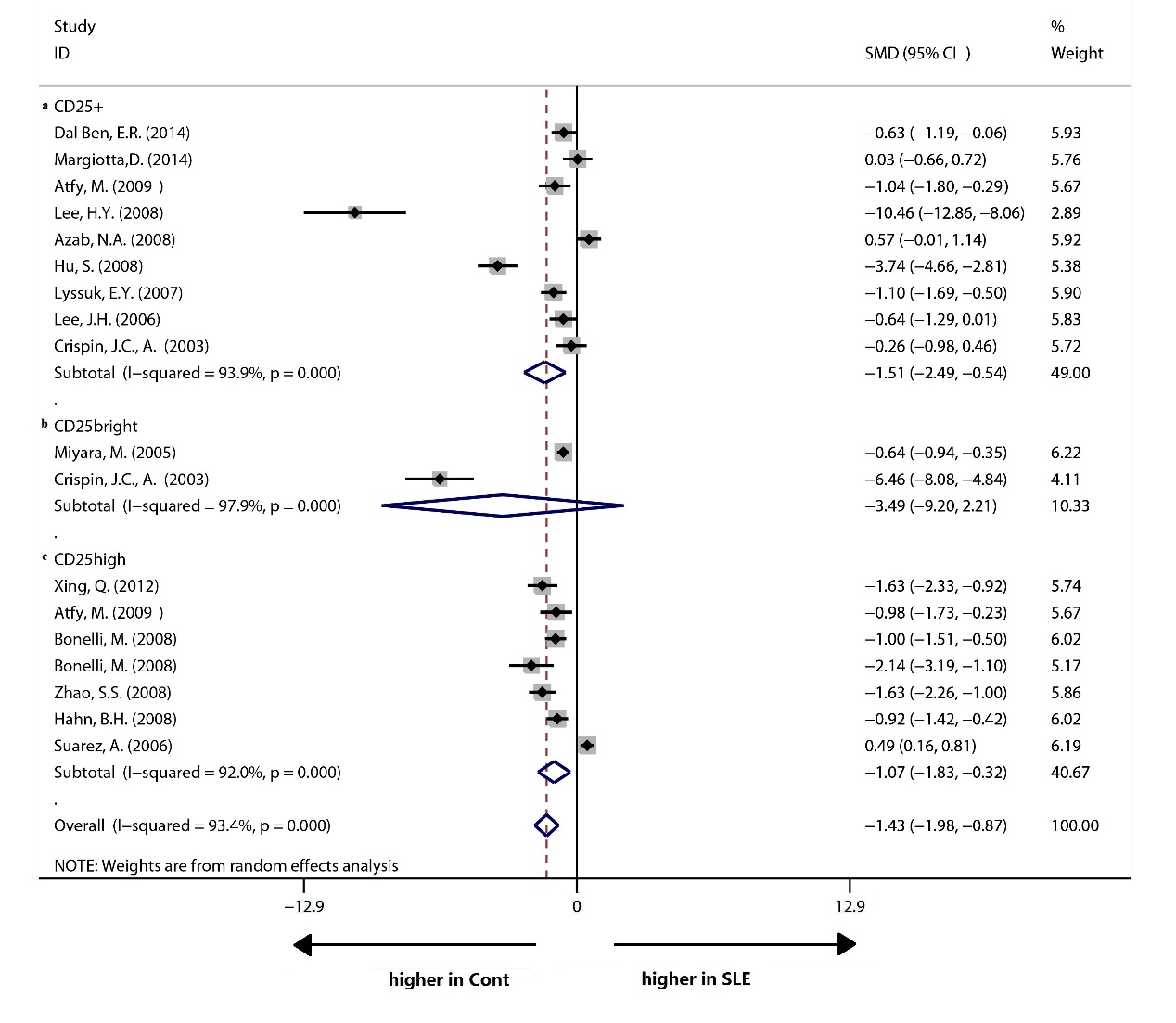


**a.** Standardized mean differences (SMDs) (the proportion of Tregs [“CD25^+^” cells] among CD4^+^ T cells in the PB of SLE patients minus that of control subjects) as estimated by meta-analysis. **b.** SMDs when Tregs were defined as “CD25^bright^” cells. **c.** SMDs when Tregs were defined as “CD25^high^” cells.

**Supplementary Fig. 2:** Forest plots of subgroups analyses of the proportion of Tregs defined as forkhead box protein P3 (FOXP3)-positive among CD4+ T cells in PB.


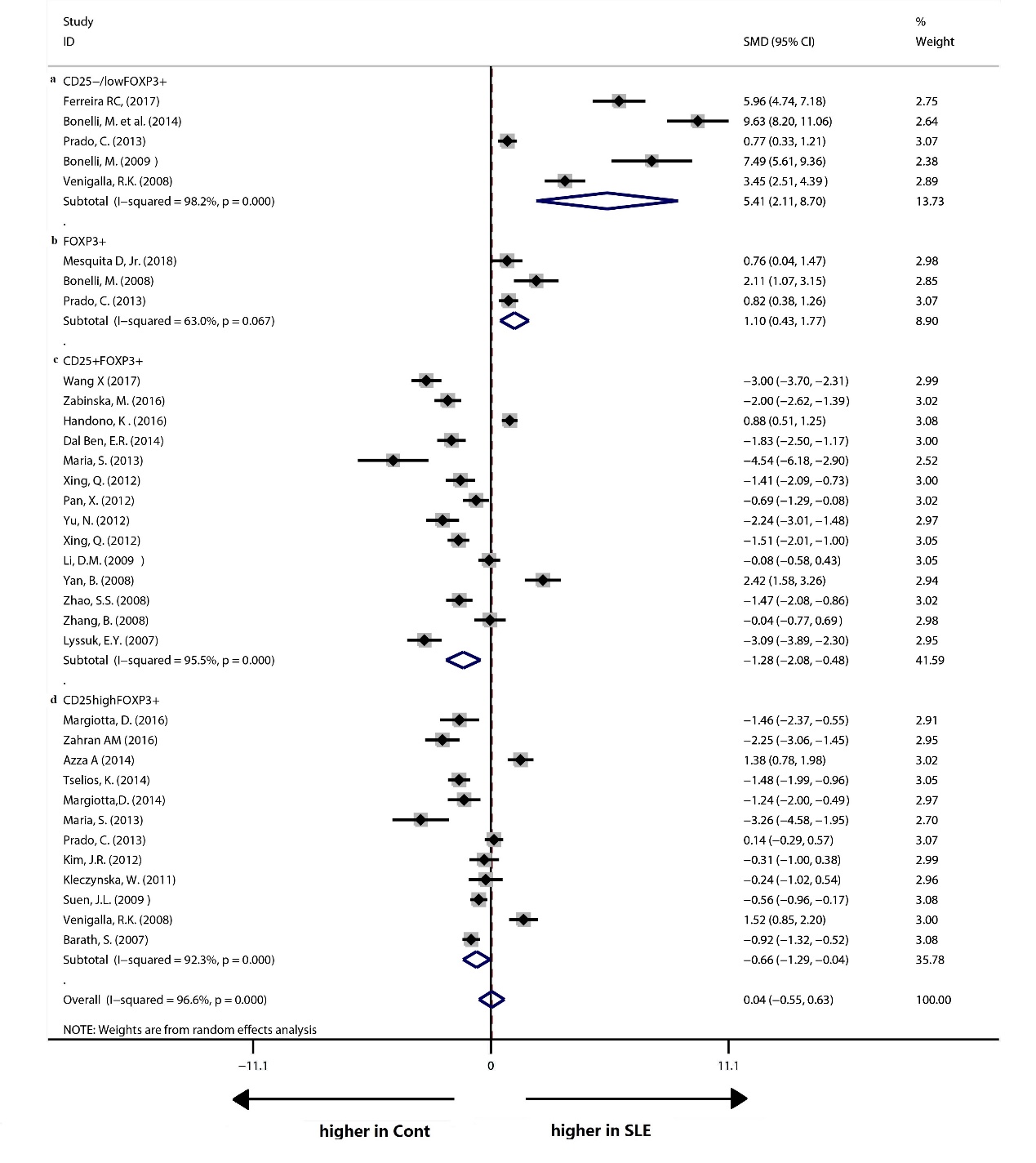


**a.** SMDs (the proportion of Tregs [“CD25^low/-^FOXP3^+^” cells] among CD4^+^ T cells in the PB of SLE patients minus that of control subjects) as estimated by meta-analysis. **b.** SMDs when Tregs were defined as “FOXP3^+^” cells. **c.** SMDs when Tregs were defined as “CD25^+^FOXP3^+^” cells. **d.** SMDs when Tregs were defined as “CD25^high^FOXP3^+^” cells.

**Supplementary Fig. 3:** Forest plots of the subgroup analyses of the proportion of Tregs that were CD127-negative among PB CD4+ T cells.


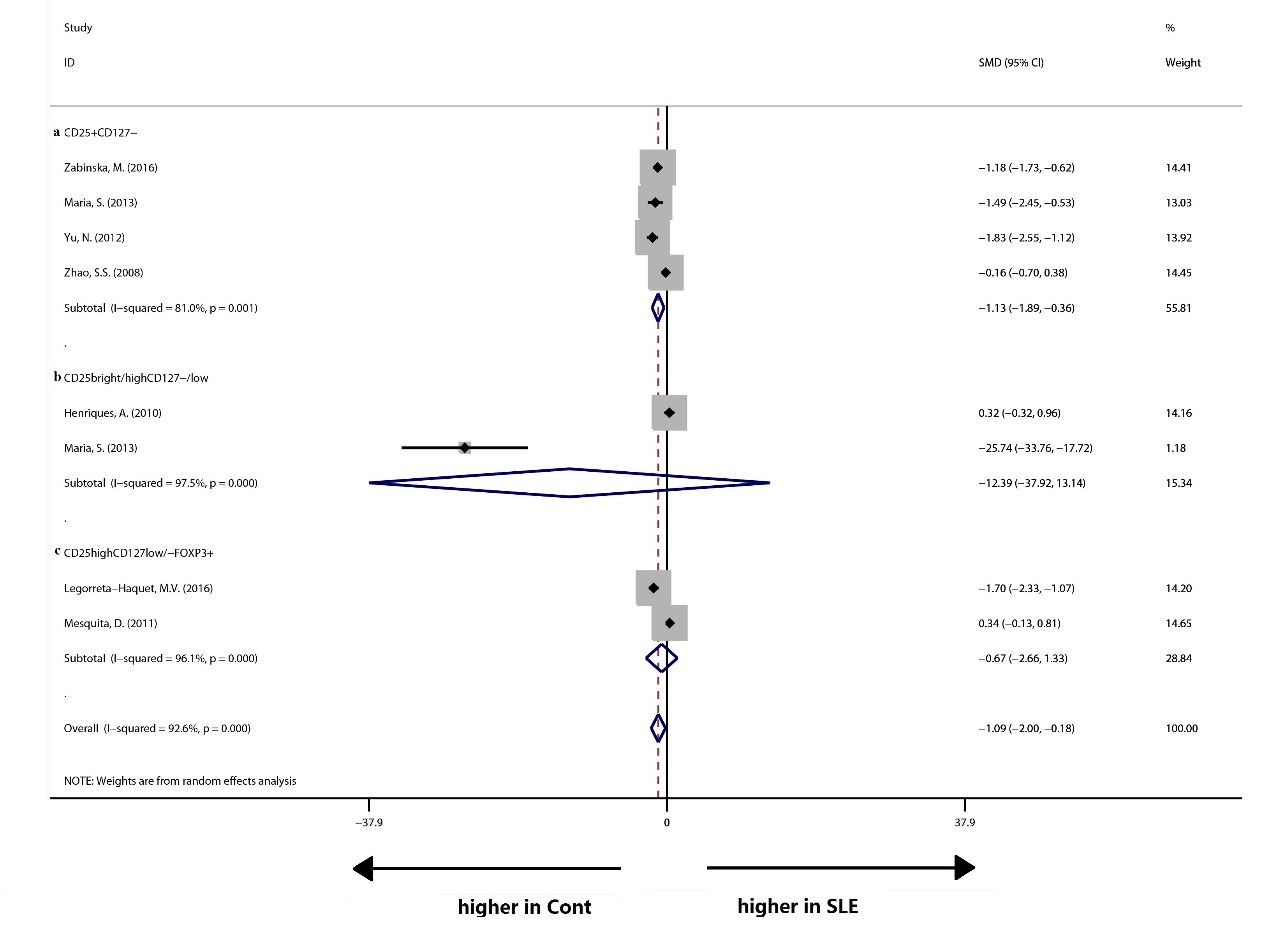


**a.** SMDs (the proportion of Tregs [“CD25^+^CD127^-^” cells] among CD4^+^T cells in the PB of SLE patients minus that of control subjects) as estimated by meta-analysis. **b.** SMDs when Tregs were defined as “CD25^high^CD127^low/-^” cells. **c.** SMDs when Tregs were defined as “CD25^high^CD127^-^” cells. **d.** SMDs when Tregs were defined as “CD25^high^CD127^low/-^FOXP3^+^” cells.

**Supplementary Fig. 4:** Funnel plot for publication bias in an association analysis of Treg proportions in PB, regardless of the Treg definitions used, between systemic lupus erythematosus (SLE) patients and healthy controls.


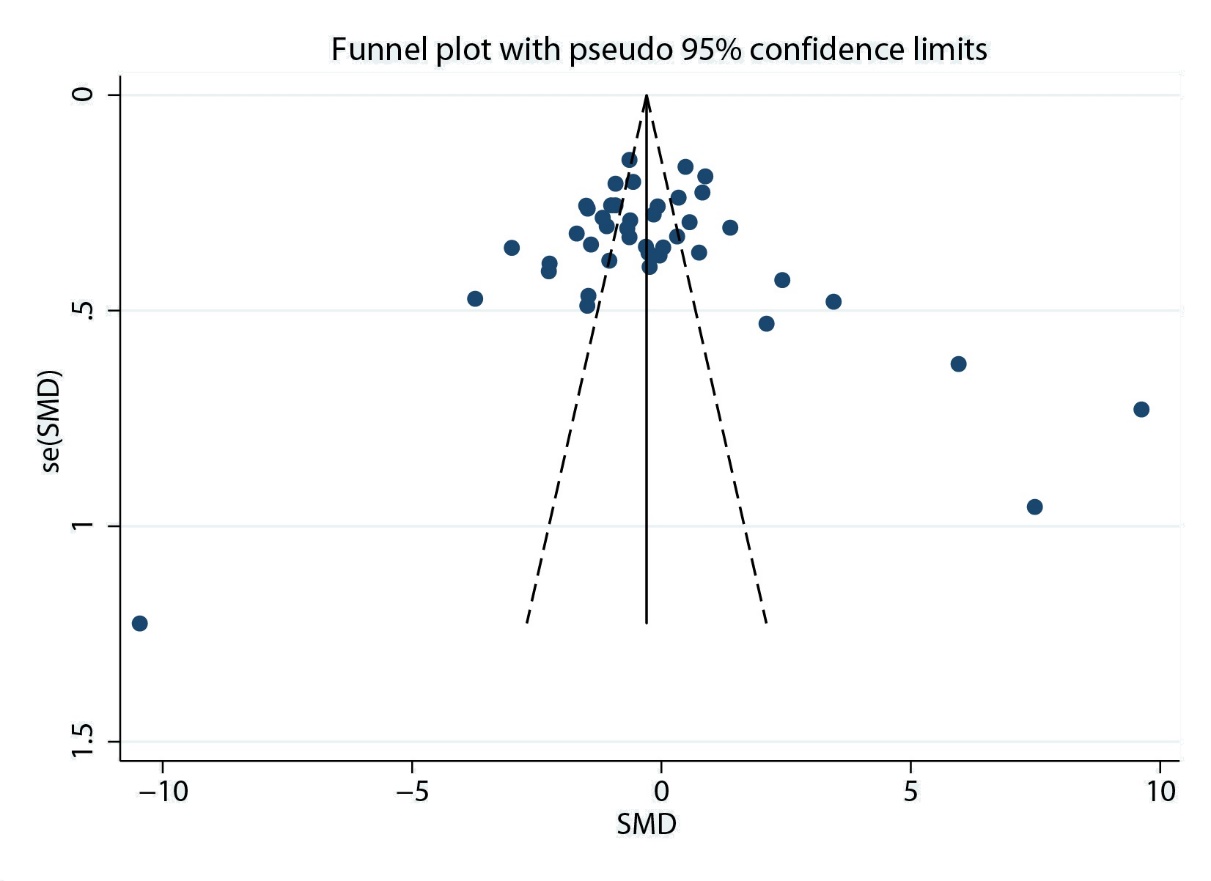


**Supplementary Fig. 5:** Funnel plot for publication bias in an association analysis of the proportion of Tregs, defined in terms of CD25-positivity (alone), among CD4+ T cells in PB.


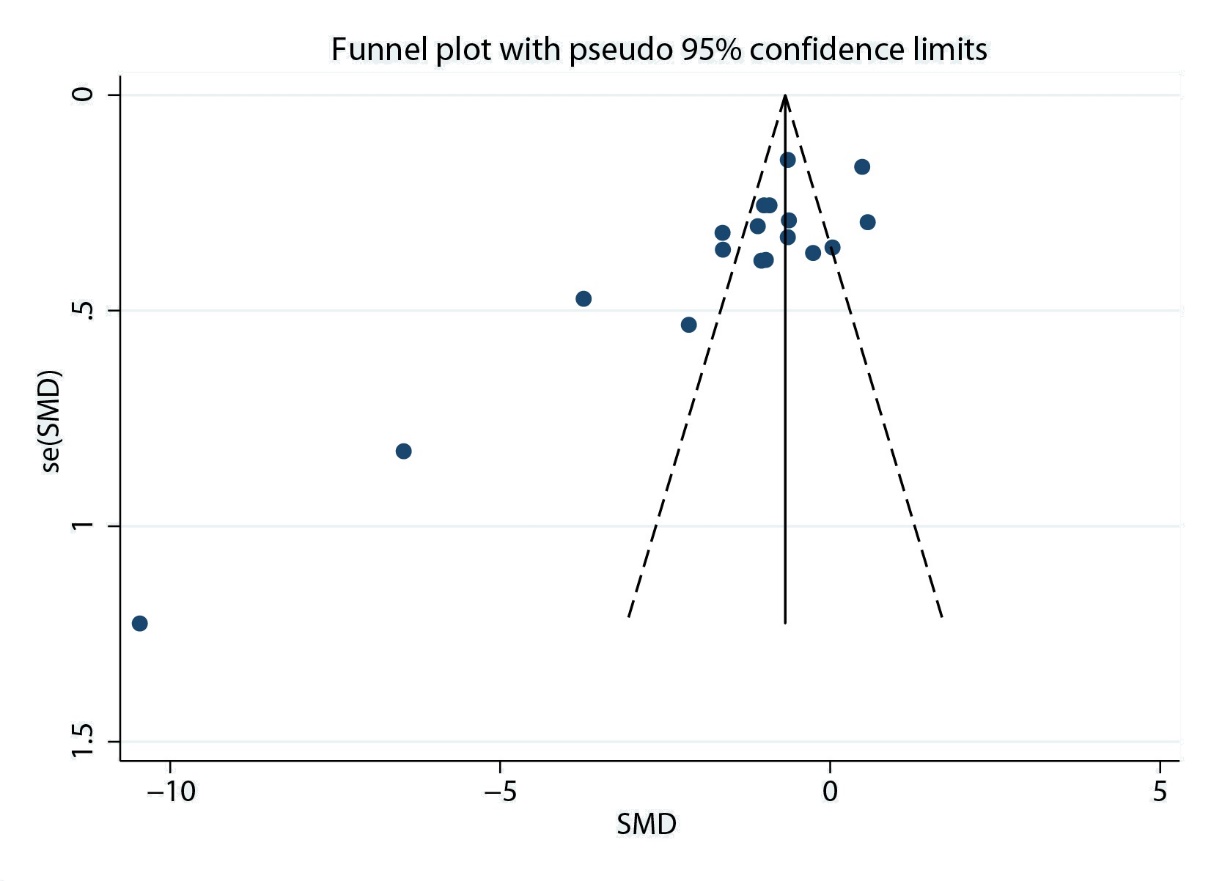


**Supplementary Fig. 6:** Funnel plot for publication bias in an association analysis of the proportion of Tregs defined as FOXP3-positive among CD4+ T cells in PB.


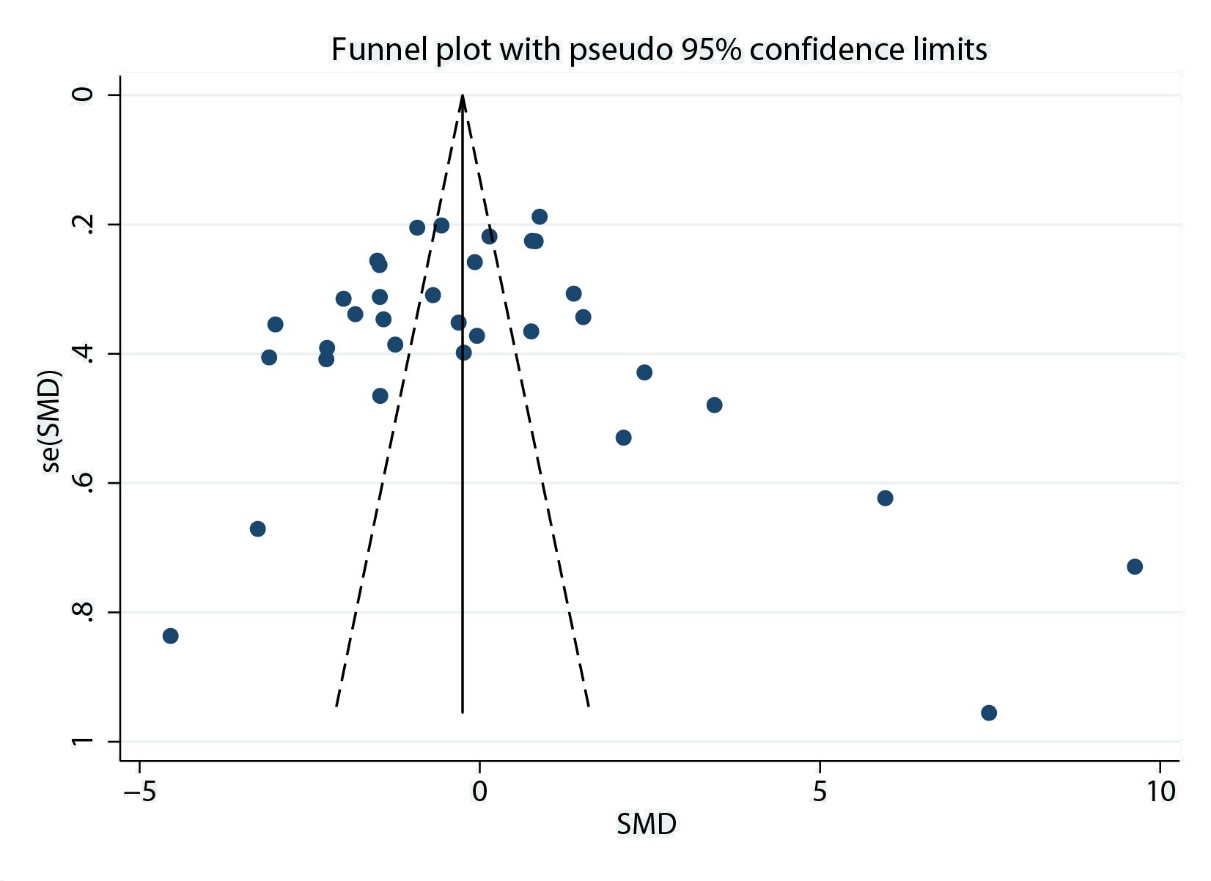


**Supplementary Fig. 7:** Funnel plot for publication bias in an association analysis of the proportion of Tregs that were CD127-negative among PB CD4+ T cells.


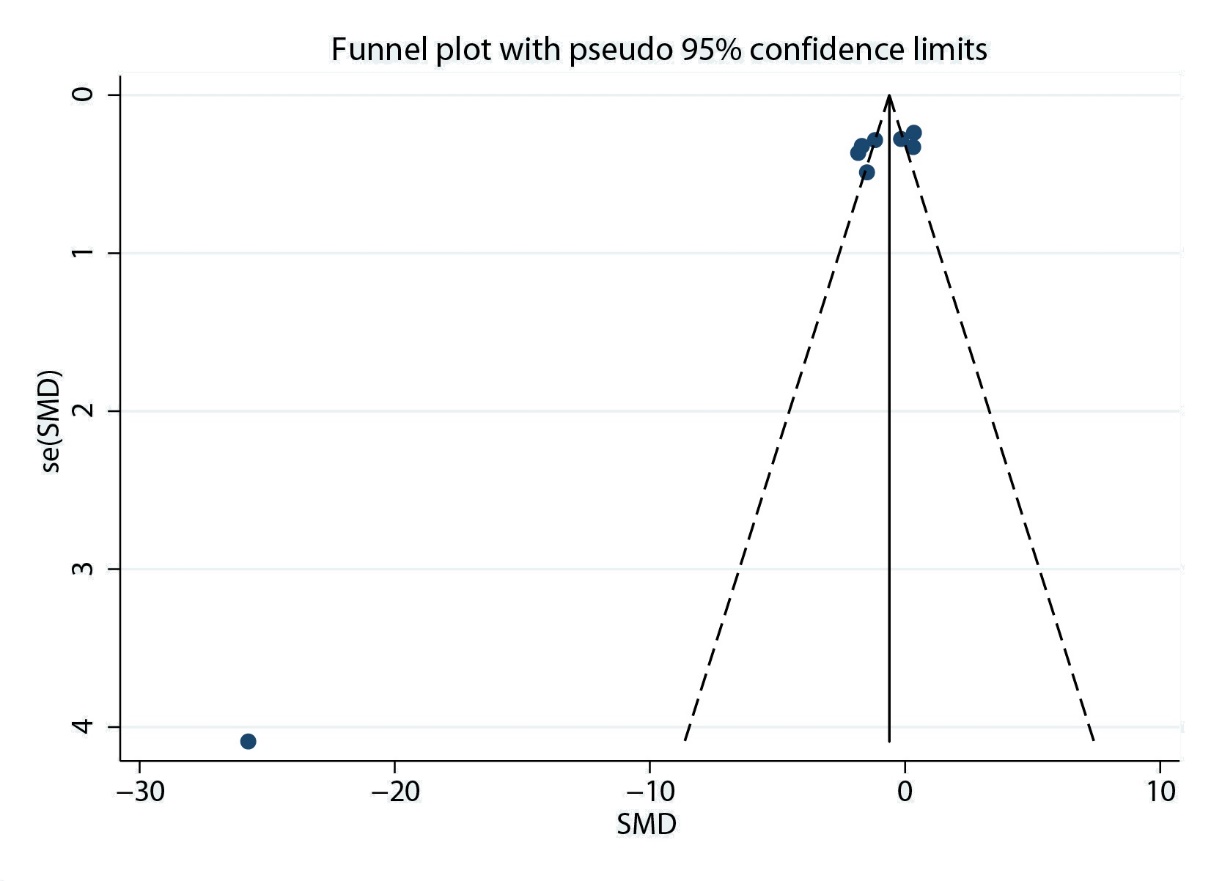

Supplement: Supplementary Materials — Supplementary Table 1: background of SLE patients in each study. Supplementary Table 2: characteristics of the included studies measuring disease activity in patients with SLE. Supplementary Figure 1: forest plot of subgroup analyses of the proportion of Tregs, defined in terms of CD25-positivity (alone), among CD4+ T cells in PB. (a) Standardized mean differences (SMDs) (the proportion of Tregs [“CD25+” cells] among CD4+ T cells in the PB of SLE patients minus that of control subjects) as estimated by meta-analysis. (b) SMDs when Tregs were defined as “CD25bright” cells. (c) SMDs when Tregs were defined as “CD25high” cells. Supplementary Figure 2: forest plots of subgroup analyses of the proportion of Tregs defined as FOXP3-positive among CD4+ T cells in PB. (a) SMDs (the proportion of Tregs [“CD25low/−FOXP3+” cells] among CD4+ T cells in the PB of SLE patients minus that of control subjects) as estimated by meta-analysis. (b) SMDs when Tregs were defined as “FOXP3+” cells. (c) SMDs when Tregs were defined as “CD25+FOXP3+” cells. (d) SMDs when Tregs were defined as “CD25highFOXP3+” cells. Supplementary Figure 3: forest plots of the subgroup analyses of the proportion of Tregs that were CD127-negative among PB CD4+ T cells. (a) SMDs (the proportion of Tregs [“CD25+CD127−” cells] among CD4+ T cells in the PB of SLE patients minus that of control subjects) as estimated by meta-analysis. (b) SMDs when Tregs were defined as “CD25highCD127low/−” cells. (c) SMDs when Tregs were defined as “CD25highCD127−” cells. (d) SMDs when Tregs were defined as “CD25highCD127low/−FOXP3+” cells. Supplementary Figure 4: funnel plot for publication bias in an association analysis of Treg proportions in PB, regardless of the Treg definitions used, between systemic lupus erythematosus (SLE) patients and healthy controls. Supplementary Figure 5: funnel plot for publication bias in an association analysis of the proportion of Tregs, defined in terms of CD25-positivity (alone), among CD4+ T ce [file 7103219.f1.docx]
